# Use of an Amplatzer ASD Occlusion Device for the Closure of an Ascending Aortic Pseudoaneurysm Presenting as Hemoptysis

**DOI:** 10.1155/2022/9809289

**Published:** 2022-02-11

**Authors:** Nicholas P. Kondoleon, Christopher Kanaan, Jonathan Hansen, Samir R. Kapadia

**Affiliations:** ^1^Department of Internal Medicine, Cleveland Clinic Foundation, 9500 Euclid Ave, Cleveland 44195, OH, USA; ^2^Department of Cardiovascular Medicine, Cleveland Clinic Foundation, 9500 Euclid Ave, Cleveland 44195, OH, USA

## Abstract

Aortic pseudoaneurysms can commonly be caused by previous thoracic surgery, trauma, and infection, quickly becoming life-threatening if ruptured. This pathology is typically asymptomatic and incidentally found on imaging; however, few cases have outlined hemoptysis as a presenting symptom for aortic pseudoaneurysms. Traditionally, management of these patients included surgical correction; however, percutaneous approaches have emerged as a safe alternative, helping to reduce the risk of morbidity and mortality associated with surgical correction. This report seeks to describe a case in which hemoptysis was the symptom unveiling the finding of a thoracic ascending aortic pseudoaneurysm and the use of an Amplatzer atrial septal defect (ASD) occlusion device as a viable option to safely resolve the disease process.

## 1. Introduction 

A pseudoaneurysm is defined as an expectoration of blood from the true lumen of a vessel, containing the connective tissue of the blood vessel and the adventitia. In contrast, a true aneurysm is defined as a progressive, nonreversible process in which all three layers of the blood vessel form an outpouching. Common causes of aortic aneurysms include atherosclerosis, infections, connective tissue disorders, autoimmune disorders, and chest trauma, among others [1]. One case report, also, describes radiation therapy as a precipitant for aortic wall weakening and rupture [2]. Pseudoaneurysms, however, are more commonly caused by previous thoracic surgery, trauma, and infection [1]. Thoracic pseudoaneurysms tend to have few symptoms and are incidentally found on chest imaging and, however, can become life-threatening quickly if ruptured. Hemoptysis is a rare presenting symptom, but may be present as a result of erosion of the surrounding airway and lung parenchyma and disruption to the surrounding bronchial arteries.

The atrial septal defect (ASD) occluder device was first introduced in 1975 by King and Mills as an alternative to traditional surgical corrections of ASDs, decreasing mortality and morbidity. Since that time, the device has evolved to become the mainstay of treatment for the correction of ASDs. Today, the Amplatzer ASD occlusion device is the most commonly used device. The device is deployed percutaneously and consists of a self-expanding double disk composed of nitinol mesh, sitting on both sides of the septal defect and occluding the defect. In recent years, the ASD occlusion device has been utilized as a solution to many other pathologies—in addition to ASD closure—including aortic pseudoaneurysms, false dissection lumens, aortocaval fistulas, and sinus of valsalva aneurysms, among others.

## 2. Case Report

A 64-year-old male presented to the hospital for hemoptysis. He had a history of coronary artery disease and had a coronary artery bypass graft (CABG) in 2004 (left internal mammary artery (LIMA)-left anterior descending (LAD), saphenous vein graft (SVG)-obtuse marginal (OM1), SVG-diagonal (Dg), and SVG-posterior descending artery (PDA)) and repeat in 2018 (LIMA-LAD and SVG-Dg patent, SVG-right coronary artery (RCA), SVG-left circumflex (LCx), and SVG-LAD). He also had a history of Hodgkin's lymphoma treated with chemotherapy and radiation that was completed in 2019, type II diabetes complicated by retinopathy, hypertension, and hyperlipidemia. The patient began experiencing hemoptysis in November 2020. This episode was followed by three more episodes: ten days from the first (lasting three days), ten days following the second episode (isolated, one time), and once in December 2020 (isolated, one time). For the past three years, he had suffered from a nonproductive cough, but only as of November, had it become productive of blood on these accounts. Following the first episode of hemoptysis, he was admitted for evaluation and reported staying for only one day following resolution of symptoms in a hospital abroad. He reported returning to the hospital in December, where he had a 5-day hospitalization with computed tomography angiography (CTA) showing a dilated thoracic aorta and treatment with “cough medicine and antibiotics.” Pictures of the hemoptysis from the patient showed blood-soaked tissues <500 mL. Other symptoms the patient endorses are chest pain radiating to back with episodes of hemoptysis, shortness of breath, and sore throat. The patient remained hemodynamically stable through his stay with stable hemoglobin.

**Figure 1:**
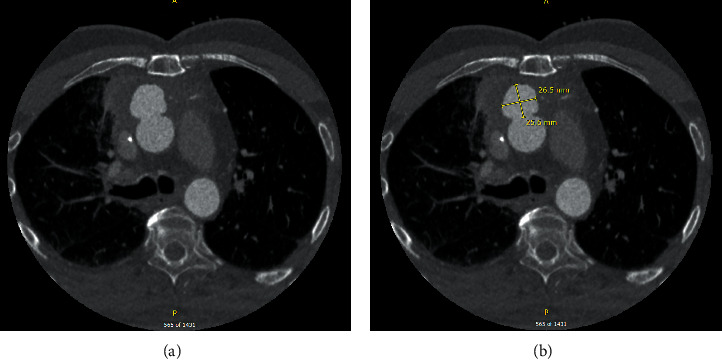
Computed tomography angiography demonstrating a large aortic pseudoaneurysm within the anterior ascending aorta.

During this admission, his echocardiogram showed an ejection fraction of 51%, grade I diastolic dysfunction, a resting wall abnormality in the RCA territory, and only trace aortic regurgitation. The patient brought CTA images from his prior admission in December. Review of the CTA revealed a linear area in the right upper lobe (RUL) representative of bronchiectasis consistent with his prior radiation therapy, a subtle enhancement in the RUL, and an aneurysmal outpouching of the thoracic ascending aorta with no appreciable communication to the RUL (Figure 1). Pulmonology was consulted for hemoptysis, and bronchoscopy with bronchoalveolar lavage was considered, but deferred following the below findings. Infectious workup and autoimmune workups were deferred due to no systemic signs of each.

**Figure 2:**
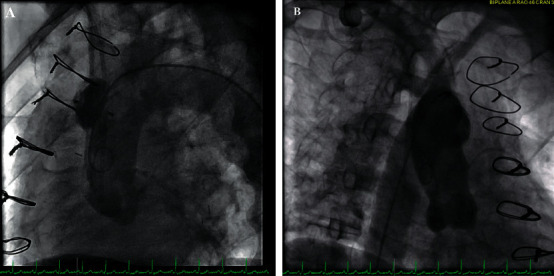
Invasive angiography with engagement of the ascending aortic arch, demonstrating a pseudoaneurysm in the anterior mid-ascending aorta. (a) LAO caudal view. (b) RAO cranial view.

The patient remained hemodynamically stable with no need for red blood cell transfusions through his admission. He was taken for a diagnostic angiography and percutaneous closure of the pseudoaneurysm on day three of his admission. The diagnostic angiography demonstrated known severe native artery disease including a chronic total occlusion of the LAD, proximal LCx, and proximal RCA, with patent grafts. Following this, the ascending aortic pseudoaneurysm was engaged identifying a pseudoaneurysm similar to the CTA findings (Figure 2). An arch aortogram was used to identify the bronchial artery, which was then engaged from the descending aorta using a left coronary bypass (LCB) catheter. This demonstrated no appreciable communication between the pseudoaneurysm and bronchial artery. An 8 Fr shuttle sheath was advanced within a 10 Fr R sheath over a Wholey wire. A 16 mm ASD Amplatzer occlusion device was then advanced over the 8 Fr shuttle and deployed to the pseudoaneurysm without complication. Angiography following deployment showed no residual flow into the pseudoaneurysm, confirming occlusion.

**Figure 3:**
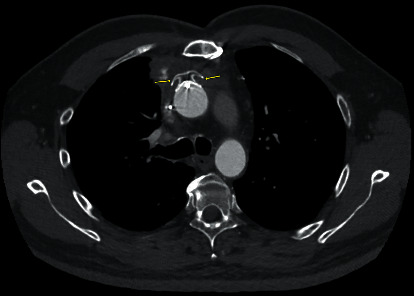
Computed tomography angiography demonstrating no overt contrast flow around a well-sealed ASD Amplatzer occlusion device within the ascending aortic anterior pseudoaneurysm one month following the procedure.

The patient was discharged four days following admission. He followed up in the cardiology clinic one month after discharge with resolution of his hemoptysis and no postintervention complications. At the time of his follow-up appointment, he had a repeat CTA showing a well-sealed plug in the anterior mid-ascending aorta with occlusion of the prior pseudoaneurysm and no overt contrast flow around the device (Figure 3). The patient then returned to his home country with continued follow-up with his home physicians.

## 3. Discussion

Few cases in the current literature describe hemoptysis as a presenting symptom for the finding of a thoracic aortic pseudoaneurysm [3–5]. In patients with recurrent hemoptysis of unknown origin, it is currently recommended to obtain a CT scan of the chest and, if unrevealing, obtain a bronchoscopy for direct visualization of the airways. In this patient, clinical presentation indicated a three-month history of recurrent, non-life-threatening hemoptysis. CT scan of the chest was revealing a pseudoaneurysm in the ascending aorta and a questionable area of bronchiectasis in the right upper lobe. Bronchoscopy was discussed, but deferred following the decision to proceed with a percutaneous closure of his pseudoaneurysm. The most common causes of recurrent hemoptysis include bronchiectasis, carcinoid tumors, AV malformations, and pseudohemoptysis. Bronchial pseudoaneurysms are also a known cause of hemoptysis; however, thoracic aortic pseudoaneurysms presenting with hemoptysis are rare. Thoracic pseudoaneurysms can often remain clinically silent; however, theorized mechanisms of hemoptysis include erosion of surrounding parenchyma as a result of compression from the pseudoaneurysm [5], alteration of the surrounding lung architecture, resulting in bronchial artery hyperplasia and tortuosity and increased susceptibility to rupture [6], formation of an aortobronchial fistula, and rupture of the pseudoaneurysm. These complications can quickly become life-threatening, specifically in the case of ruptured pseudoaneurysms. In the case of an aortobronchial fistula, though rare, left-to-right shunting can manifest as heart decompensation or cardiogenic shock, lending to challenges in care due to hemodynamic instability [7].

Traditionally, surgery was the recommended approach for the correction of thoracic pseudoaneurysms; however, it carries a risk of 7%–41% mortality [8]. As a result, percutaneous, endovascular approaches have emerged as a viable alternative to traditional therapy. In the past decade, case reports have described the successful treatment of aortic pseudoaneurysms using ASD occlusion devices, percutaneously [8–12]. Additionally, Toušek et al. described a case in which an endovascular closure of a pseudoaneurysm complicated by an aortobronchial fistula helped to alleviate the hemodynamic challenges that would have been faced during a surgical correction alone due to left-to-right shunting [7]. Use of endovascular techniques for pseudoaneurysm closure has been shown to be feasible and safe, helping to avoid complications that arise with high-risk surgical repair. In this case, the patient's history of a CABG and radiation therapy predisposed him to the formation of a thoracic pseudoaneurysm. Use of a 16 mm ASD Amplatzer occlusion device deployed percutaneously proved to be a viable and safe option for the patient, resulting in successful occlusion with no residual contrast flow around the device on CTA one month following the procedure.

## 4. Conclusion

Although rare, hemoptysis has been described in few case reports as the presenting symptom of thoracic pseudoaneurysms due to erosion of the surrounding airway and lung parenchyma, increased fragility and susceptibility to rupture of surrounding bronchial arteries, formation of aortobronchial fistulas, or rupture. In this case, treatment with a 16 mm ASD Amplatzer occlusion device resulted in resolution of the patient's recurrent hemoptysis and avoidance of high-risk surgical repair and its complications.

## Data Availability

No data were used to support this study.
